# Influence of Microstructure on Dynamic Mechanical Behavior and Damage Evolution of Frozen–Thawed Sandstone Using Computed Tomography

**DOI:** 10.3390/ma16010119

**Published:** 2022-12-22

**Authors:** Junce Xu, Hai Pu, Ziheng Sha

**Affiliations:** 1 State Key Laboratory for Geomechanics and Deep Underground Engineering, China University of Mining and Technology, Xuzhou 221116, China; 2 College of Mining Engineering and Geology, Xinjiang Institute of Engineering, Urumqi 830091, China

**Keywords:** computed tomography, F–T action, microstructure, dynamic mechanics, damage evolution

## Abstract

Frost-induced microstructure degradation of rocks is one of the main reasons for the changes in their dynamic mechanical behavior in cold environments. To this end, computed tomography (CT) was performed to quantify the changes in the microstructure of yellow sandstone after freeze–thaw (F–T) action. On this basis, the influence of the microscopic parameters on the dynamic mechanical behavior was studied. The results showed that the strain rate enhanced the dynamic mechanical properties, but the F–T-induced decrease in strength and elastic modulus increased with increasing strain rate. After 40 F–T cycles, the dynamic strength of the samples increased by 41% to 75.6 MPa when the strain rate was increased from 75 to 115 s^−1^, which is 2.5 times the static strength. Moreover, the dynamic strength and elastic modulus of the sample were linearly and negatively correlated with the fractal dimension and porosity, with the largest decrease rate at 115 s^−1^, indicating that the microscopic parameters have a crucial influence on dynamic mechanical behavior. When the fractal dimension was increased from 2.56 to 2.67, the dynamic peak strength of the samples under the three impact loads decreased by 43.7 MPa (75 s), 61.8 MPa (95 s), and 71.4 MPa (115 s), respectively. In addition, a damage evolution model under F–T and impact loading was developed considering porosity variation. It was found that the damage development in the sample was highly related to the strain rate and F–T damage. As the strain rate increases, the strain required for damage development gradually decreases with a lower increase rate. In contrast, the strain required for damage development in the sample increases with increasing F–T damage. The research results can be a reference for constructing and maintaining rock structures in cold regions.

## 1. Introduction

In cold regions, F–T weathering can lead to disasters such as the collapse of rock structures, landslides, and mudslides [1,2]. The microstructural remodeling caused by ice crystal pressure is the main reason for altering the mechanical behavior of rocks [3]. In addition, rock structures in cold environments are often affected by dynamic loads [4]. For example, the slopes of open-pit mines are not only affected by frost, but also susceptible to dynamic loads such as blasting and mechanical disturbances [5]. Therefore, studying microstructural changes under F–T action in response to dynamic loads is of great practical importance for the construction and maintenance of rock structures in cold regions.

The dynamic mechanical properties of rocks in cold regions have been extensively studied. The Split Hopkinson Pressure Bar (SHPB) system is one of the important devices for performing dynamic tests [6]. For instance, Xu et al. [7] investigated the dynamic mechanical properties of sandstone under F–T action using the SHPB system and found that the dynamic strength and modulus decreased exponentially with F–T damage, and developed a model to predict the decrease in strength and modulus. Li et al. [8] studied the influence of microstructure on the dynamic mechanical characteristics of the sandstone under F–T action using SHPB and nuclear magnetic resonance (NMR) systems, and pointed out that large pores are the key factor affecting the dynamic strength. Liu et al. [9] performed SHPB tests on the rock with different F–T cycles and concluded that the more severe the F–T damage, the greater the degree of rock fragmentation. In addition, Xu et al. [10] investigated the energy evolution of the sandstone during impact and suggested that F–T damage increases the dissipation energy during impact, which in turn increases the macroscopic fragmentation of the sample. Zhang et al. [11] investigated the effects of F–T action and impact loading on the brittleness of rock using the SHPB system and showed that the higher the number of F–T cycles, the lower the dynamic brittleness index, and concluded that increased porosity was the main reason. The above work mainly studied the macroscopic dynamic mechanical behavior of rocks induced by F–T action and did not quantify the effects of F–T action on microstructure. However, it is difficult to consider the damage mechanism of rocks only from the macroscopic point of view [12]. In fact, the evolution of the microstructural properties of rocks is the main cause of the changes in their macroscopic mechanical behavior [13]. Therefore, the study of the effects of F–T cycles on the dynamic behavior of rocks should begin at the microscopic level [14].

In recent years, many researchers have applied various methods, including acoustic emission (AE), scanning electron microscopy (SEM), NMR, and CT, to reveal the decay characteristics and damage mechanisms of rocks from a microscopic perspective. Fang et al. [15] used the SEM method to observe rocks subjected to different F–T cycles. They found that F–T damage is often due to pre-existing defects and that the initiation and propagation of cracks correlates closely with the original pore structure of the rock. Cheng et al. [16] reported the influence of F–T action on the microscopic pores of the rock based on NMR, and concluded that F–T damage promotes the development of various pore sizes, especially medium and large pores in the rock. Xu et al. [17] analyzed the promotion of the rock damage process using the AE method and found that the AE signals were more active in the sample with higher F–T cycles. However, the SEM method can only observe the deterioration of the sample surface, and the AE method is disturbed by environmental interference, while the NMR method cannot detect the morphological features of the pores; moreover, it is difficult to relate the changes in the parameters to the macroscopic mechanical properties [12]. The CT technique, as a non-destructive testing approach, can analyze the rock degradation process caused by F–T action from a microscopic point of view [18]. Yang et al. [19] evaluated the evolution of soft rock microstructure under F–T action using the CT technique and adopted the CT number as a variable to define F–T damage. Fan et al. [20] conducted real-time CT tests on sandstones with different freezing temperatures and noticed that the damage pattern of sandstones below −10 °C tended to be stable. In addition, Maji et al. [21] performed a 3D reconstruction of the sandstone based on the CT technique to study the microstructural deterioration of the samples with different F–T damage. Li et al. [1] combined CT and visualization techniques to analyze F–T damage and argued that inherent defects in rocks are the main factor for F–T damage. In general, the internal structural deterioration from F–T action is responsible for the variations in the macro-mechanical behavior of rocks [22]. However, these studies are limited to the F–T damage mechanisms without connecting changes in microscopic parameters to mechanical behavior, exceptionally dynamic mechanical behavior.

To this end, this study investigates the effects of microscopic parameters such as porosity, permeability, and fractal dimension on the dynamic mechanical behavior of sandstone through CT scanning and SHPB tests. Based on the CT results, a damage model of the sample under F–T and impact loading was developed. This work can serve as a reference for the construction and maintenance of slopes in open pit mines in cold areas.

## 2. Materials and Experimental Methods

### 2.1. Materials

**Figure 1:**
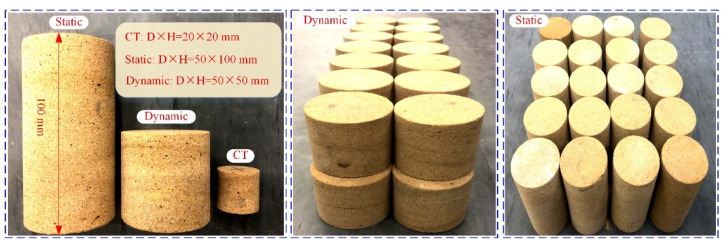
Diagram of static and dynamic samples.

**Figure 2:**
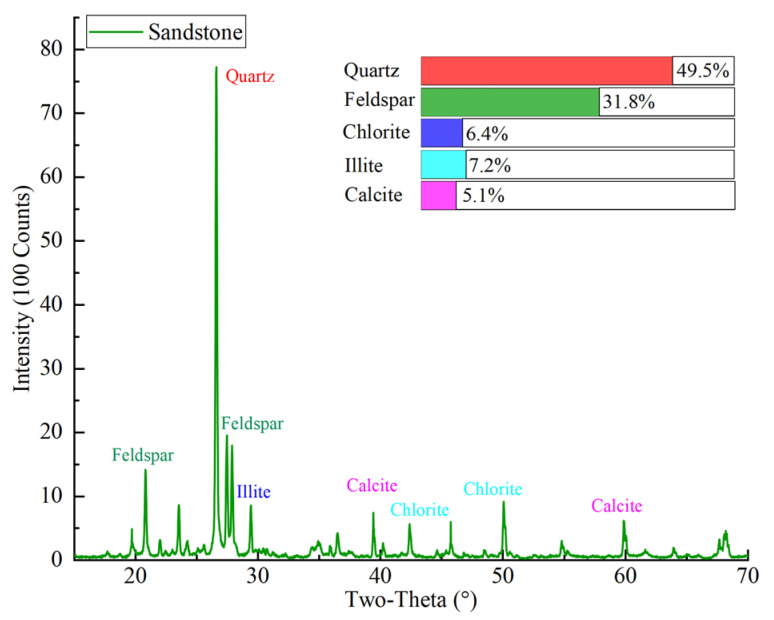
Results of X-ray diffraction test.

The yellow sandstone for the work was sampled from the slope of an open pit mine in Urumqi, China. The selected yellow sandstone is a common sedimentary rock in the Xinjiang region of China. At the same time, the Xinjiang region belongs to the seasonally cold areas and the rock structures are susceptible to F–T weathering. Homogeneous blocks of the sandstone were selected to machine cylindrical samples with a diameter of 50 mm according to ISRM recommendations. Two aspect ratios were machined, namely, 1 for dynamic tests and 2 for static tests (see Figure 1). The lateral surfaces of the samples need to be vertical and smooth, with no significant structural surfaces. In addition, both ends of the samples were ground to a tolerance of 0.02 mm [8]. Then, the dry density and longitudinal wave velocity of the samples were measured to select samples with similar wave velocity and density for this study. A total of 54 samples were selected for the dynamic tests and divided into three groups for the F–T tests. Moreover, 21 samples with a length of 100 mm were selected to conduct static tests. To maximize spatial resolution, samples with a diameter of 20 mm were chosen for the CT scanning tests. Three static samples were selected for the basic physico-mechanical tests. The results are listed in Table 1. The main mineral composition of the sandstone was determined by X-ray diffraction tests (Bruker AXS, Karlsruhe, Germany, product model D8-A), and the results are presented in Figure 2.

### 2.2. Experimental Methods

#### 2.2.1. F–T Tests

**Figure 3:**
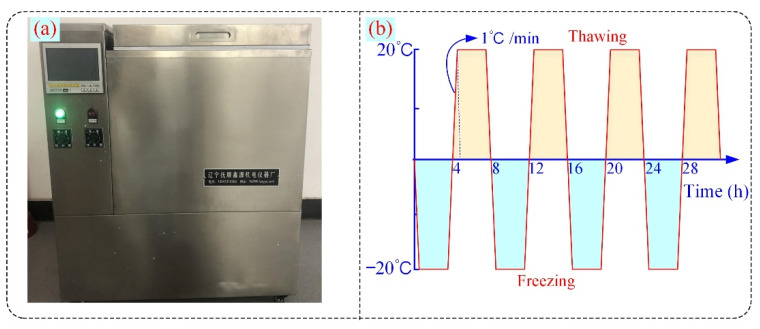
F–T chamber (**a**) and cyclic temperature setting (**b**).

To accelerate the weathering process, a rapid F–T chamber (Liaoning Fushun Instrument Co., Ltd., Fushun, China, product model QDR-50) was used in this study (see Figure 3). The chamber can operate in a temperature range of −30 to 80 °C with a control accuracy of ±0.5 °C. The cyclic F–T number in this study was set to 0, 5, 10, 20, 30, and 40 times according to the temperature variations in the cold areas. According to ASTM D5312, the F–T process was performed with a cycle of −20 °C freezing for 4 h and 20 °C thawing for 4 h. In this work, only saturated sandstone was tested for F–T cycles.

#### 2.2.2. CT Scanning Tests

**Figure 4:**
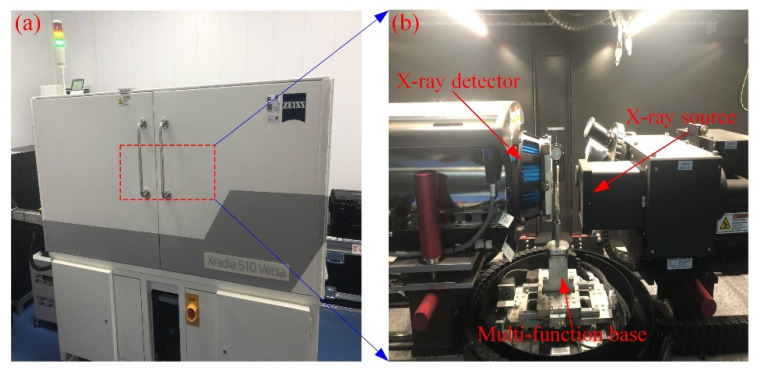
Zeiss Xradia 510 Versa high-resolution CT system (**a**) and interior (**b**).

As shown in Figure 4, a Zeiss Xradia 510 Versa high-resolution 3D X-ray microscope was used to perform CT scans of the samples with different F–T cycles. The instrument mainly consists of an X-ray source, an X-ray detector, and a multi-function base. The instrument is imaged by passing the target sample through the radiation source and measuring the attenuation energy to extract the density data of the object. In this procedure, the sample is rotated 360° to obtain thousands of vertical 2D images in all directions, which are reconstructed into approximately thousands of horizontal 2D images by computer processing [23]. When these 2D images are stacked sequentially, they can be displayed as a 3D image. In the binarization technique, pixels below the threshold are called voids, and pixels above the threshold are called rock matrix, and in turn, the voids and rock are separated [24]. That is, the pore skeleton can be extracted and used to analyze the length or connectivity of the pores. The structure of the pores can also be visualized in 3D, allowing quantification of the size, orientation, and interconnectivity of the pores in the rock. The scanning time for each sample was about 2 h. The main acquisition parameters of the system during scanning are listed in Table 2, where the current and voltage parameters can influence the number of X-rays and the intensity of penetration [25]. The CT scan of the entire rock sample yielded about 1100 horizontal images with 1024 × 1024 pixels in 16-bit grayscale. Since the CT scan is a non-destructive technique, the scan does not affect the subsequent F–T tests.

In this study, raw data from CT tests were processed using Avizo (2019.1 version) software (Thermo Fisher Scientific Co. Shanghai, China), which provides noise reduction, threshold segmentation, binarization, and 3D reconstruction of CT images [26]. By identifying the pores and matrix of the rock sample, Avizo can calculate the porosity of the sample using Equation (1), where *V* and *V*_d_ denote the volume of the sample and the defect volume, respectively. Also, the permeability of the sample can be determined using the XLabSuite module integrated into Avizo. Note that the XLabSuite module uses Darcy’s law (Equation (2)) to calculate permeability and can only estimate the connected pores in the 3D diagram [26].

(1) $$P=\frac{V_{d}}{V}$$

(2) $$k=\frac{Q\mu L}{A\Delta P}$$

where *k* is the absolute permeability, μm^2^; *Q* is the flow rate of water, μm^3^/s; *μ* is the viscosity of the liquid, which for water at room temperature is 0.001 Pa·s; *L* is the length of the seepage channel, μm; *A* is the outlet area of the liquid, μm^2^; *p* is the pressure difference between import and export, Pa, which in Avizo is 10 kPa by default.

#### 2.2.3. Static Tests

**Figure 5:**
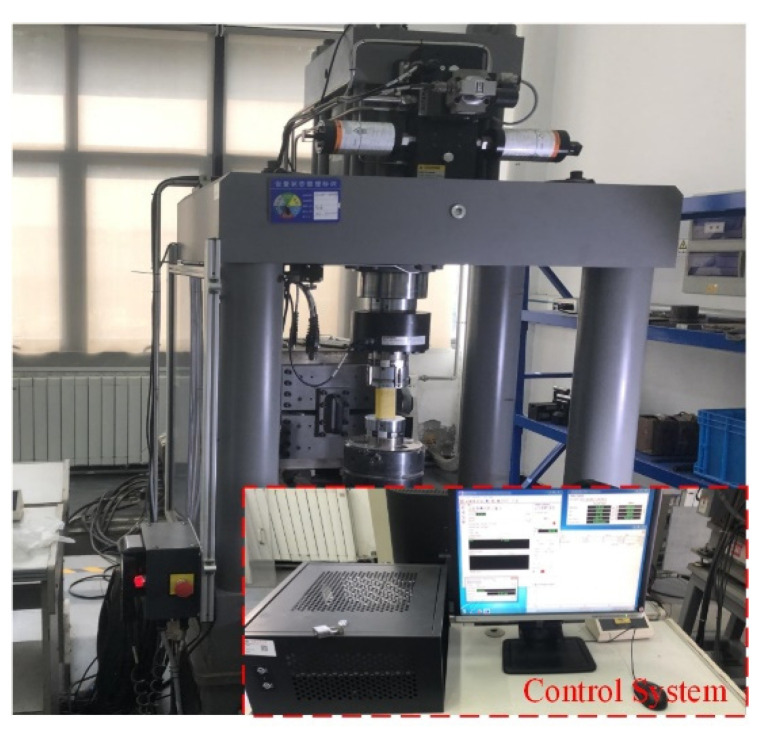
MTS816 experimental system.

In this study, static uniaxial compression tests were performed on the freeze–thawed samples using the MTS816 system (MTS USA, Inc. Minneapolis, MN, USA), as shown in Figure 5. The MTS816 system measured the axial displacement of the samples via a linear variable displacement transducer (LVDT), recorded the axial load, and determined the stress–strain relationship of the specimens. To better control the loading process, the displacement control method was used during the test and the displacement rate was set to 0.1 mm/min to meet the requirements of quasi-static loading.

#### 2.2.4. Dynamic Impact Tests

**Figure 6:**
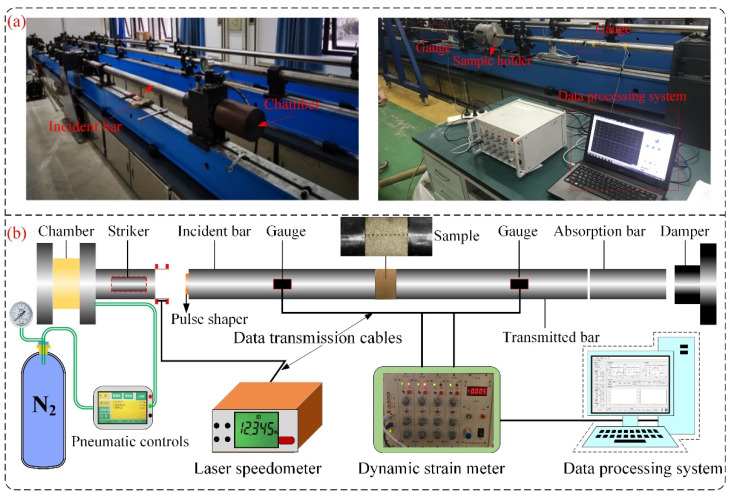
SHPB experimental system (**a**) and detailed schematic diagram (**b**).

As shown in Figure 6, impact tests were performed on sandstone samples using a SHPB system. The system mainly consists of four parts: an impact load system, a compression bar system, a data acquisition system, and a damping system. In the SHPB system, both the striker and the bar are 50 mm in diameter and are made of Cr40 with a P-wave velocity of 5400 m/s, and yield strength and elastic modulus of 800 MPa and 208 GPa, respectively. To obtain a suitable waveform, a circular rubber pad (D × H = 10 mm × 2 mm) was selected as the pulse shaper. Before the test, molybdenum disulfide was applied to the ends of the samples as a lubricant to reduce the friction effect [27]. When the high-pressure gas drives the striker against the incident bar, an elastic compression wave is generated. A portion of the compression wave can propagate through the sample to the transmitted bar. Based on the 1D stress wave propagation principle, the three-wave analysis method was adopted to calculate the dynamic loads *P*_1_ and *P*_2_, strain rate, and strain at both ends of the sample (Equation (3)) [5]. In addition, to ensure dynamic load equilibrium at both ends of the samples during the test, the stress waves at both ends need to be verified before the test [28]. After all samples had undergone the specified F–T cycles, dynamic tests with three impact loads were performed with the SHPB system to obtain the macroscopic dynamic mechanical properties of the yellow sandstone.

(3) $$\{\begin{array}{l} P_{1}(t)=A_{r}E_{0}\left[\varepsilon_{i}(t)+\varepsilon_{r}(t)\right]\;,\;P_{2}(t)=A_{r}E_{0}\varepsilon_{t}(t)\\ \dot{\varepsilon }(t)=\frac{C_{0}}{L}[\varepsilon_{i}(t)-\varepsilon_{r}(t)-\varepsilon_{t}(t)]\\ \varepsilon (t)=\int_{0}^{t}\dot{\varepsilon }(t)dt=-2\frac{C_{s}}{L}\int_{0}^{t}\varepsilon_{r}(t)dt \end{array}$$

where $\varepsilon_{i}(t)$, $\varepsilon_{r}(t)$, and $\varepsilon_{t}(t)$ refer to the incident, reflected, and transmitted waves, respectively; *A*_r_ is the ratio of the cross-sectional area of the bar to the sample; *E*_0_, *C*_0_, and *L* are the elastic modulus and P-wave velocity of the bar and the length of the sample, respectively.

## 3. Experimental Results and Discussion

### 3.1. 3D Reconstruction

**Figure 7:**
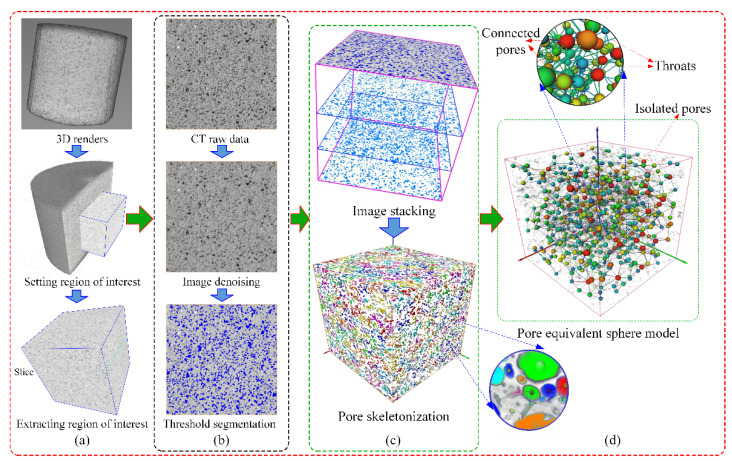
CT image processing and reconstruction process. (**a**) Region of interest; (**b**) 2D image processing; (**c**) 3D reconstruction; (**d**) PNM model.

As shown in Figure 7, slice data from the CT were imported into Avizo for 3D reconstruction. Compared to 2D images, 3D images can provide a visual assessment of the spatial structure and reflect the development of F–T damage in the rock. Since the artifacts at the sample edges could not be effectively avoided, and the data volume of the whole rock sample was large, the central cubic region was cropped out for 3D reconstruction and analysis with Avizo (Figure 7a). To avoid the influence of volume on the experimental results, the representative elementary volume (REV) was determined semi-automatically using the Auto-refresh tool in Avizo. This command can calculate the volume fraction of pores or matrix starting from multiple voxels with an arbitrary segmentation threshold, where the smallest volume at which the volume fraction tends to a stable value is the REV. Ultimately, a volume of 750 × 750 × 750 voxels was chosen, corresponding to the actual physical size of the 15 × 15 × 15 mm^3^ cube. The Register Image command was used for spatial alignment to ensure the same spatial position of the rock sample under different F–T conditions [26]. It is worth mentioning that all CT results were matched in grayscale to minimize the impact of the CT scanning process on the experimental results.

In addition, the median filtering method was chosen to reduce noise points and artifacts caused by factors such as the environment and the rock itself, thus smoothing the distribution of greyscale values in the region (Figure 7b). This method can remove almost all of the noise without affecting the original image. At the same time, filtering the images can improve the image quality to speed up the processing and visualization of the experimental data from CT. In this study, the interactive segmentation algorithm in Avzio was used to perform threshold segmentation of pores and structures in CT images. To determine a suitable segmentation threshold, the actual porosity of the samples was first tested using the mercury intrusion method [12]. Then, the porosity of the 3D samples was calculated using Equation (1) based on the grayscale distribution features of the CT images, and the segmentation threshold T = 4076 between the rock matrix and the pores was determined using the backstepping method. Because greyscale matching was performed for the CT images, only the segmentation threshold T was required to complete the segmentation of all data. Finally, the 3D reconstruction model of the sandstone subjected to F–T action was developed (Figure 7c). In order to statistically capture the data of the defects in the 3D model, such as the pore equivalent diameter, throat length, etc., the PoreNetworkModeling (PNM) method in Avizo was used in this study, as shown in Figure 7d. Here, the pore is equated to a sphere using the equivalent sphere method, while the microcrack is equated to a throat [26]. The 3D images show that the sandstone pores are divided into isolated and interconnected pores, some of which are connected by throats.

### 3.2. Effect of F–T on Microscopic Parameters

**Figure 8:**
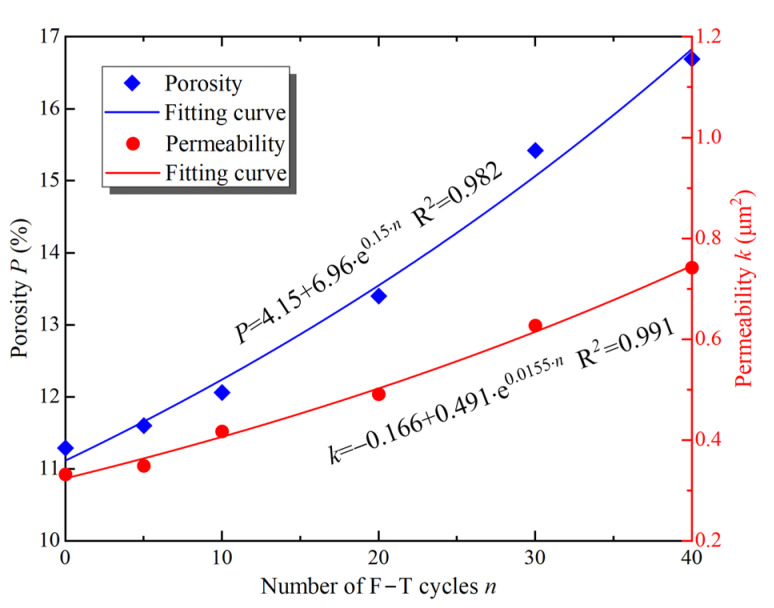
Changes in porosity and permeability of the sample with F–T cycles.

As shown in Figure 8, based on the 3D reconstruction model, the variation of porosity and permeability of the samples was calculated using Equations (1) and (2). Porosity and permeability are closely related to the nature of the rock and are important parameters to describe the pore network in the rock. As seen in Figure 8, porosity and permeability of the samples increase with F–T cycles, and the exponential function can describe the changes of the parameters well. After 40 F–T cycles, the porosity increased to 16.25%, which is 1.3 times higher than the samples without F–T action. Similarly, the permeability of the samples subjected to 40 F–T cycles increased by 128% to 0.742 μm^2^. The increased porosity and permeability of the sample provides favorable conditions for migration of water and heat during freezing or thawing, which in turn promotes damage to the rock by F–T action. As a natural material, rocks indeed contain a large number of pores and microcracks. The pore water turns into ice at low temperatures, causing volume expansion. When the pore ice does not have enough space to expand freely, frost heaving will occur [4]. At the same time, the pores and microcracks are gradually enlarged by the frost heaving force. During thawing, pore water flows through the microcracks into other pores, and at this time, the microcracks play the role of seepage channels. For this reason, the microcracks are also called throats. In addition, the water in the rock promotes the expansion of the pores and microcracks through lubrication, erosion, hydrolysis, and F–T action [29]. Meanwhile, some isolated pores develop into interconnected pores through throats. Therefore, the porosity and permeability of the sample simulated with XLabSuite increase with the F–T action. In addition, the increase in porosity and permeability also means an increase in defects in the samples, which leads to a deterioration of the mechanical properties under load.

**Figure 9:**
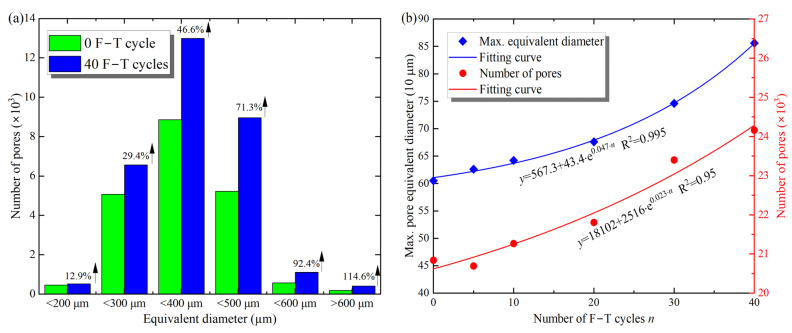
Distribution of the pore equivalent diameters (**a**) and the maximum and total number of pores (**b**) with F–T cycles.

Additionally, the pore parameters of the sample, such as pore equivalent diameter and throat length, were determined using the PNM model. The pore diameters were divided into six ranges according to their sizes, i.e., 0–200 μm, 200–300 μm, 300–400 μm, 500–600 μm, and above 600 μm, and the results are shown in Figure 9a. After 40 F–T cycles of the sample, the total number of pores increased from 20,832 to 24,160 by about 15.98%, and the number of pores in all ranges showed an increasing trend. According to the distribution of pore diameter values, the minimum increase in the number of pores (<200 μm) was 12.9%, and the maximum (>600 μm) was 114.6%. In general, the larger the pore equivalent diameter, the more significant the percentage growth in the number of pores after F–T action. In other words, the larger the pore diameter, the more susceptible it is to F–T action, and the increase in the number of pores implies an increase in F–T weathering damage. Moreover, Figure 9b shows the maximum pore diameter and pore number with the F–T action, and the increase is consistent with the pattern of change in porosity and permeability, i.e., an exponential increase. Compared to the samples without F–T action, the maximum pore diameter of the sample increased from 605 μm to 856 μm after 40 F–T cycles, an increase of about 41.4%. The pores also showed an exponential growth pattern, increasing by 12.3% to 23,408 after 30 F–T cycles. The increase in equivalent diameter and pores reflects the increase in pore volume, expressed macroscopically as an increase in porosity in Figure 8. Overall, the increase in the number of pores and the equivalent diameter of the sample caused the expansion and development of microcracks, which in turn caused macroscopic damage to the mechanical properties.

**Figure 10:**
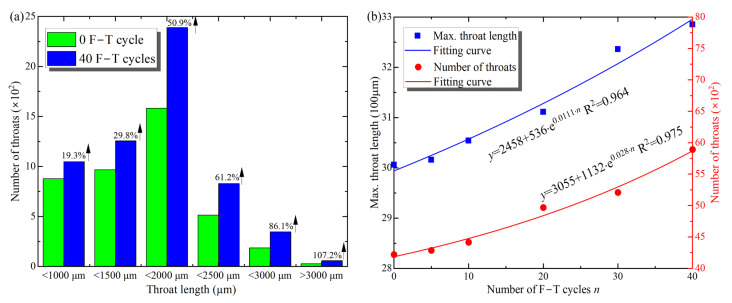
Distribution of the lengths (**a**) and the maximum lengths and total number (**b**) of throats with F–T cycles.

Furthermore, Figure 10 shows the range of length distribution, number, and maximum length of throats with F–T cycles. From Figure 10a, the number of throat lengths shows an increasing trend in all ranges, and the greater the throat length, the more pronounced the increase. For example, the number of throats longer than 3000 μm increased by 107.2% after 40 F–T cycles, whereas the number of throats shorter than 1000 μm increased by only 19.3%, consistent with the pore growth trend. Besides, the changes in maximum length and the total number of throats with F–T action are shown in Figure 10b, and the exponential function can describe this increasing trend. After 40 F–T cycles, the maximum throat length increased from 3006 to 3285 μm, an increase of 9.3%, and the total number of throats increased by 37.4% from 4221 to 5893. Similar to the changes in pore parameters, the length and number of throats better reflect the microcrack development inside the sample. Therefore, the increase in the number and length of the throats indicates the cumulative macroscopic damage caused by the F–T action on the rock.

In summary, the internal damage in rocks, such as pores or microcracks, tends to increase with increasing F–T cycles, and especially larger defects increase more. The freezing of pore water in the sample at low temperature is closely related to the pore diameter, and the equation for the freezing point drop of pore water is as follows [18]:

(4) $$T_{fre}=-273\frac{2\sigma }{r\rho L}$$

where *r* is the equivalent diameter of the ice crystals, ρ is the ice density, σ is the pore interface tension, and *L* is the latent heat of the phase change. It is seen that the smaller the diameter of the ice crystals, the lower the freezing point of the pore water. Thus, as the temperature is below 0 °C, the supercooled water is in the small pores. In other words, the smaller the pore volume, the less it is affected by the cyclic F–T effect [29], which can also be seen in Figure 9 and Figure 10, i.e., the smaller the pore volume, the lower the increase. Two main mechanisms cause F–T weathering. The first is the volume expansion mechanism, which causes a 9.05% increase in volume when the water (>91%) in pores, cracks, joints, and other voids turns to ice at below 0 °C [2]. When the pore water content exceeds 91%, it exerts a frost heaving pressure on the pore wall. At this point, in an ideal environment, the maximum stress of 207 MPa can be applied to the matrix, but in practice, a pressure of 10 to 100 MPa is applied to the matrix [14]. The second one is generated by the movement of unfrozen water in the rock. In this study, the volume expansion was considered significant due to the cyclic F–T performed on the saturated samples.

### 3.3. Effect of F–T on Fractal

**Figure 11:**
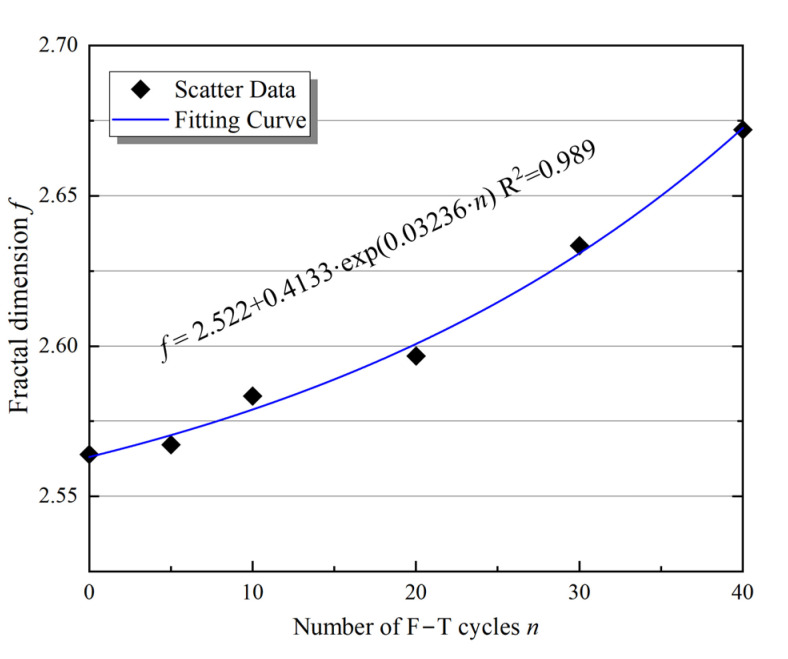
Variation of 3D fractal dimension of samples with different F–T cycles.

To describe the influence of F–T action on the internal microstructural features of the sample, fractal theory, which responds to the spatial occupancy of complex shapes, was introduced as a measure of irregular, nonlinear shapes [30]. Therefore, the fractal theory is used to quantify the evolution of microstructure in rocks under F–T cycles using 3D CT technology. The fractal dimension is the most important parameter to describe the fractal. In general, the fractal dimensions mainly include the Hausdorff dimension, the assignment dimension, the box dimension, and the similarity dimension [31]. The box dimension is widely used because of its clear physical meaning [12]. In this study, the box dimension was used; that is, the 3D pore model of the sample was placed in a series of cubes with a grid side length *ε*. The number of grids containing pores or cracks in the cubes was *P*(*ε*), and a series of *P*(*ε*) was obtained by continuously adjusting the dimensions of the small grids. Then, the slope of the regression line is obtained using double logarithmic coordinates for *P*(*ε*) and *ε*. The fractal dimension *f*, which corresponds to the edge length *ε* of the grid, is therefore given in Equation (5). Using the fractal dimension module in Avizo, the fractal dimension *f* of the central region was determined under F–T action [26]. The results are shown in Figure 11.

(5) $$f=\underset{\varepsilon \to 0}{\lim}\frac{\ln P(\varepsilon )}{\ln(1/\varepsilon )}=-\underset{\varepsilon \to 0}{\lim}\frac{\ln P(\varepsilon )}{\ln(\varepsilon )}$$

**Figure 12:**
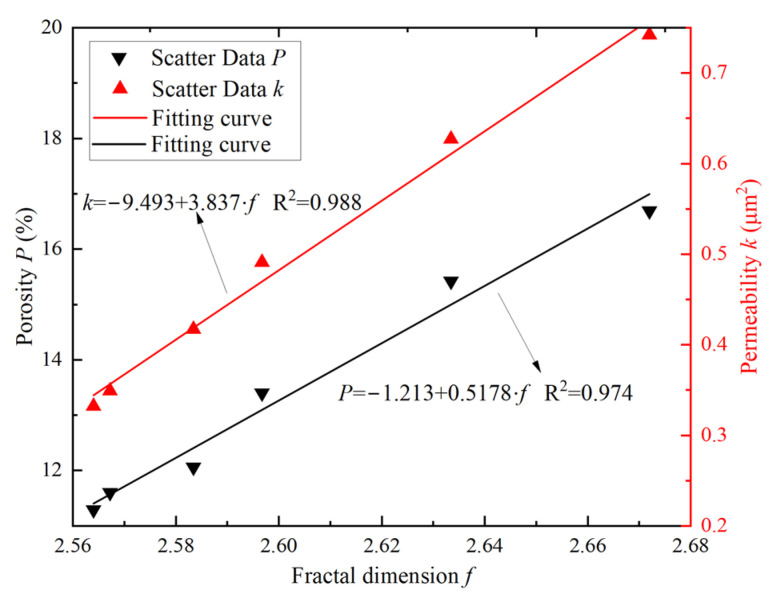
Changes in porosity and permeability with fractal dimension of the sample under the F–T action.

As seen in Figure 11, the 3D fractal dimension of the samples ranged from 2.57 to 2.70, and the variation of the fractal dimension corresponded to the exponential function, which was consistent with the analytical results of porosity, permeability, and pore parameters. The greater the cyclic F–T damage, the larger the fractal dimension of the sample and close to 3. After 40 F–T cycles of the sample, the fractal dimension increases by 4.1%, from 2.574 to 2.679. During the test, the microstructure of the sample (pores or microcracks) gradually develops due to the F–T action, and the spatial distribution becomes more complex, suggesting that the pores are interconnected by throats to increase the spatial occupancy capability. The larger the fractal dimension, the greater the F–T damage to the internal microstructures of the sample. Thus, it is reasonable to characterize the damage to the sandstone microstructure by the fractal dimension. In addition, Figure 12 shows the relationship between the fractal dimension *f* and the porosity *P*, and permeability *k* of the samples under different cyclic F–T action. 

From Figure 12, the porosity and permeability of the samples are positively correlated with the fractal dimension. This is consistent with the variation of porosity, permeability, and pore parameters with the F–T cycles. Natural rock is a fractal with obvious fractal features such as porosity and permeability [32]. Hence, the larger the fractal dimension, the greater the variability of the microstructure, and the greater the porosity and permeability of the sample [12]. In other words, the larger the fractal dimension of the sample, the faster pores and microcracks (throats) develop, resulting in more significant damage to the internal structure of the sample and a more pronounced permeability phenomenon. At the same time, the increase in porosity and permeability provides convenient conditions for water and heat migration during freezing and thawing [33]. As the number of F–T cycles increases, the expansion effect of the ice gradually increases, resulting in a more rapid development of the internal structure (pores and throats) of the sample and a more complex spatial distribution, while the pores are interconnected by throats and have an increased ability to occupy space, so that the internal microstructure of the sample is increasingly damaged by the F–T action. Therefore, the fractal dimension can quantitatively describe the dynamic evolution process of the sample microstructure and effectively characterize the development of F–T damage. Since porosity and fractal dimension are positively correlated, porosity is also an effective parameter to describe F–T damage.

### 3.4. Macroscopic Dynamic Mechanical Properties

**Figure 13:**
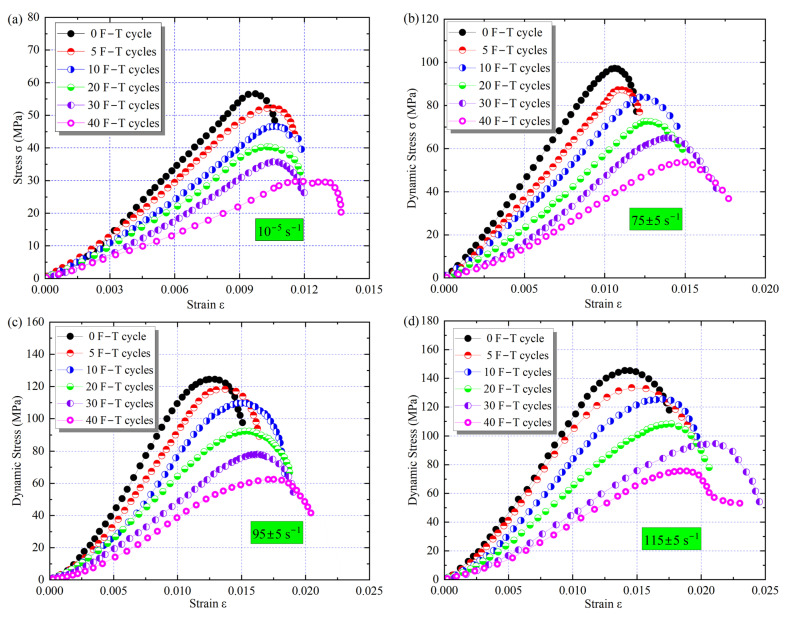
Stress–strain behavior under F–T action: (**a**) Static; (**b**) 75 ± 5 s^−1^; (**c**) 95 ± 5 s^−1^; (**d**)115 ± 5 s^−1^.

**Figure 14:**
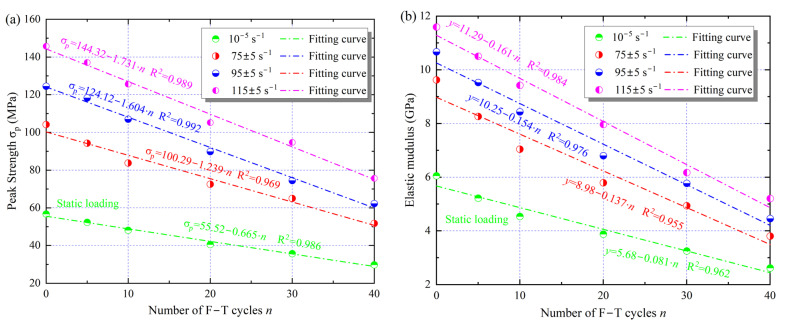
Variations of peak strength (**a**) and elastic modulus (**b**) of samples with cyclic F–T action.

To investigate the effect of changes in microscopic parameters on the mechanical behavior of the samples, a series of static and dynamic tests were performed using an MTS816 and an SHPB test system. The stress–strain curves of the samples with different cyclic F–T action are shown in Figure 13. In the static tests, the peak strength of the samples decreased with the F–T damage. However, the ultimate deformation capacity (peak strain) exhibited an increasing trend, implying that the cyclic F–T damage resulted in a decrease in the bearing capacity and deformation resistance of the rock. For example, after 10 F–T cycles, the peak strength decreased by 14% to 46.2 MPa, while the peak strain increased by 15% to 1.125%. Compared with the static test, there was a strengthening effect of strain rate, i.e., the peak strength and peak strain increased with loading strain rate. When the strain rate was increased from about 75 s^−1^ to 115 s^−1^, the dynamic strength of the sample with 20 F–T cycles increased from 65 to 105 MPa, corresponding to 2.1 and 2.7 times the static peak strength, respectively. The strength and tangential elastic modulus under different loading conditions are presented in Figure 14. Here, the slope of the straight line segment in the stress–strain curve is used as the elastic modulus [27].

As shown in Figure 14, the peak strength ($\sigma_{P}$) and elastic modulus of the sample gradually decrease with the increase of F–T cycles, which is opposite to the variation of the microscopic parameters. Moreover, the linear function can well describe the changes in the strength and elastic modulus of the sandstone, and the decrease rate is positively related to the strain rate level. The peak strengths of the samples after 40 F–T cycles decreased by 52.44, 62.28, and 73.05 MPa for the three impact loads, corresponding to 1.94, 2.31, and 2.72 times the static decrease, respectively, while the changes in the elastic modulus and peak strength of the samples were similar. Instead of static loading, the dynamic mechanical behavior of sandstone is therefore more sensitive to cyclic F–T damage.

In general, F–T damage results from the interaction of capillary mechanisms, crystallization pressure mechanisms, hydrostatic pressure mechanisms, and volume expansion mechanisms [29]. Each of these mechanisms is closely associated with the properties of pores and microcracks. Due to the different chemical potentials, the water in the pores nucleates first and then freezes, while the water in the throats is supercooled and plays a role in recharging the pores [34]. As a result, the continuous increase of frost heaving force causes the pores to be connected by the throats, leading to F–T damage such as wedge cracking [14]. Under static loading, damage in the rock, such as pores and throats, reduces the bearing area, resulting in changes in mechanical behavior, with pores playing an important role due to their large volume [35]. However, under impact loading, the stress waves propagating in the rock tend to activate the throats near the pore tips, resulting in much more damage development in the rock than under static loading [6]. Moreover, the hysteresis of rock deformation with respect to impact stress waves during dynamic loading leads to strain rate strengthening effects. The dynamic mechanical behaviors of the sandstone under cyclic F–T action are more sensitive to the changes in microscopic parameters. Therefore, it is necessary to connect microscopic parameters and dynamic mechanical properties.

### 3.5. Macro-Micro Properties Connection

**Figure 15:**
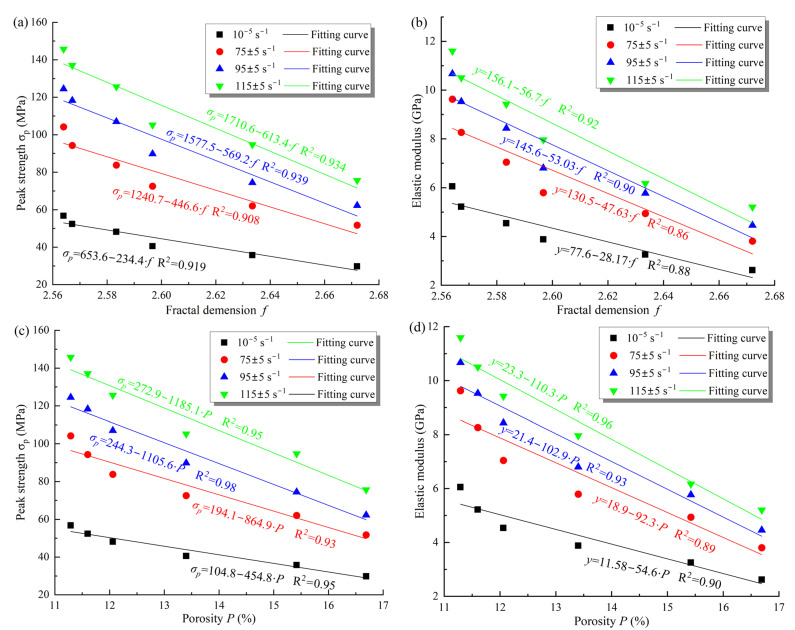
Connection between mechanical properties and microscopic parameters of samples. (**a**) Peak strength vs. fractal dimension; (**b**) elastic modulus vs. fractal dimension; (**c**) peak strength vs. porosity; (**d**) elastic modulus vs. porosity.

In this study, the relationship between microscopic parameters such as fractal dimension, and porosity and macroscopic mechanical parameters is shown in Figure 15. From that, the dynamic peak strength and elastic modulus decrease linearly with the fractal dimension and porosity of the samples under different loading conditions. Meanwhile, the rate of decrease correlates positively with the strain rate, which is consistent with the changes in mechanical properties during the F–T process. For example, when the strain rate increased from 75 s^−1^ to 115 s^−1^, the decrease rate in the dynamic elastic modulus of the samples increased from 92.3 to 110.4 with increasing porosity, which is 2.02 times the decrease rate under static loading. Similarly, the decrease in dynamic strength with decreasing porosity was 1.9, 2.4, and 2.6 times higher, respectively, than the static strength for the three loading rates. Moreover, when the fractal dimension f was increased from 2.56 to 2.67, the peak strength decreased with increasing strain rate by 52.4 MPa, 62.3 MPa, and 69.4 MPa, respectively, compared with a decrease of 26.9 MPa at static loading. Zhou et al. [36] observed the effects of water saturation times on rock microstructure using SEM and found that the mechanical properties of rock were closely related to the fractal dimension; Li et al. [1] investigated the effects of porosity on the mechanical behavior of rock during F–T cycles using the CT technique and reached conclusions similar to those in this paper. The cyclic F–T action alters the internal structure of the rock by increasing the number, and size of pores and throats, and removing some of the cement [37]. As a result, the cement strength and the cohesion between the mineral particles and the skeleton are gradually weakened, increasing internal defects and damage. At the same time, the increase in porosity and fractal dimension means an increase in the complexity of the internal structure of the rock [31]. As seen in Figure 15, the complexity and inhomogeneity of the internal structure determine the degradation of the mechanical properties, so the microstructure of the sample is the key to influence the dynamic mechanical behavior. Therefore, the variation of microstructural parameters needs to be considered in the damage evolution of the rock under impact loading.

## 4. Damage Evolution under F–T and Impact Loading

### 4.1. F–T Damage D_n_

From a macroscopic viewpoint, the response of the dynamic mechanical behavior can represent the rock damage [12]. Based on this, the elastic modulus was selected to describe the variable *D*_n_ for the F–T damage.

(6) $$D_{n}=1-\alpha \cdot \frac{E_{n}}{E_{0}}$$

where *D*_n_ represents the damage index of the sample caused by F–T action; *E*_0_ refers to the initial elastic modulus; *E*_n_ indicates the dynamic elastic modulus of the sample after n F–T cycles.

Considering that the elastic modulus of the sample is obtained only from the linear section of the curves, it is not sufficient to fully reflect the F–T damage. Moreover, the random distribution of defects, the pore volume, and the degree of crack penetration in the sample significantly affect the damage process [1]. Therefore, the change in the effective bearing volume of the rock is introduced as an improvement factor α in the damage definition.

(7) $$\alpha =\frac{V_{e}}{V}=\frac{V_{e}}{V_{e}+V_{d}-V_{\mathrm{di}}}$$

where *V*_e_ denotes the effective bearing volume (matrix volume) of the rock; *V* is the apparent bearing volume, which is composed of two main components: one is the matrix volume, and the other is the defect volume (pores and throats), also known as initial defects considering the various defects in the rock itself [2]. To remove the initial defects from the new damage induced by F–T cycles, the initial volume of defects *V*_di_ in the rock is subtracted from *V*. In the initial state of the sample, when *V*_di_ is equal to *V*_d_, the value of *D*_n_ is 0, and as *V*_d_ increases, the difference between *V*_d_ and *V*_di_ gradually increases, which means that the effect on *D*_n_ is more than obvious. Therefore, considering the defect volume, the damage factor *D*_n_ is as follows:

(8) $$D_{n}=1-\frac{V_{e}}{V_{e}+V_{d}-V_{\mathrm{di}}}\cdot \frac{E_{n}}{E_{0}}$$

**Figure 16:**
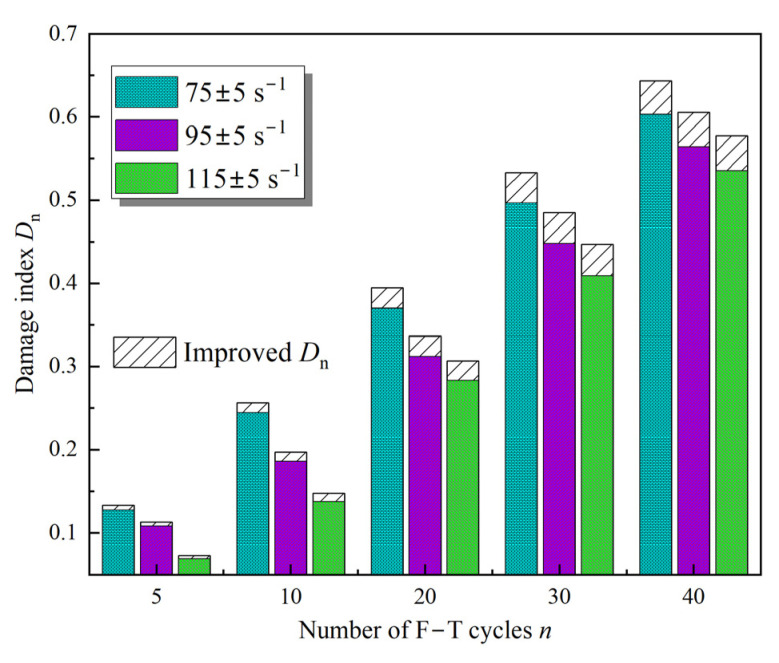
Variation of damage index *D*_n_ with different F–T cycles.

According to Figure 7c, the apparent volume and defect volume of the sample can be determined using the 3D visualization results. As shown in Equation (1), the porosity variation of the sample was calculated based on the defect volume and apparent volume [26]. Therefore, the damage index *D*_n_ of the sample was calculated using Equation (8), as presented in Figure 16. From this, it is evident that the improvement factor increases the *D*_n_, which is due to the fact that the value of α gradually decreases to less than 1. Moreover, for the same F–T cycles, the strain rate is negatively correlated with the F–T damage, i.e., the larger the strain rate, the smaller the value of *D*_n_.

### 4.2. Damage D_m_ Evaluation

The following assumptions are made to characterize the evolution of damage in the rock during dynamic loading: (1) at the macroscopic level, the rock is an isotropic, homogeneous material; (2) the microunits exhibit linear elasticity before damage; and (3) the microunits strength obeys a two-parameter Weibull distribution with a probability density function of *P*(*F*) [38]:

(9) $$P(F)=\frac{m}{F_{0}}{\left(\frac{F}{F_{0}}\right)}^{m-1}\exp[-{\left(\frac{F}{F_{0}}\right)}^{m}]$$

Following the strain strength theory, the parameter *F* can be substituted by the strain *ε* [6]. Therefore, Equation (9) can be written as follows:

(10) $$P(\varepsilon )=\frac{m}{\varepsilon_{0}}{\left(\frac{\varepsilon }{\varepsilon_{0}}\right)}^{m-1}\exp[-{\left(\frac{\varepsilon }{\varepsilon_{0}}\right)}^{m}]$$

where *ε*_0_ is scale parameter; *m* refers to the shape parameter.

If the total number of microunits is N and n units fail under impact loading, the ratio *n*/*N* is defined as *D*_s_ [39]:

(11) $$D_{s}=\frac{n}{N}=\frac{N\int_{0}^{\varepsilon }P(\varepsilon )d\varepsilon }{N}=1-\exp[-{\left(\frac{\varepsilon }{\varepsilon_{0}}\right)}^{m}]$$

As a result, the stress–strain relationship under dynamic loading was determined using the strain equivalence hypothesis [6]:

(12) $$\sigma =E_{0}\varepsilon (1-D_{s})$$

where *E*_0_ refers to the initial elastic modulus. Considering the effect of F–T action on the elastic modulus, Equation (12) can be expressed as follows:

(13) $$\sigma =\alpha E_{n}\varepsilon (1-D_{s})$$

Further, Equation (13) can be expressed as:

(14) $$\sigma =E_{0}(1-\frac{E_{0}-\alpha E_{n}}{E_{0}})\varepsilon (1-D_{s})=E_{0}\varepsilon (1-D_{n})(1-D_{s})$$

Thus, bringing Equations (8) and (11) into Equation (14), the damage *D*_m_ evolution equation for the sample under impact loading and F–T damage is as follows:

(15) $$D_{m}=D_{n}+D_{s}-D_{n}D_{s}=1-\frac{V_{e}}{V_{e}+V_{d}-V_{\mathrm{di}}}\frac{E_{n}}{E_{0}}\exp[-{\left(\frac{\varepsilon }{\varepsilon_{0}}\right)}^{m}]$$

**Figure 17:**
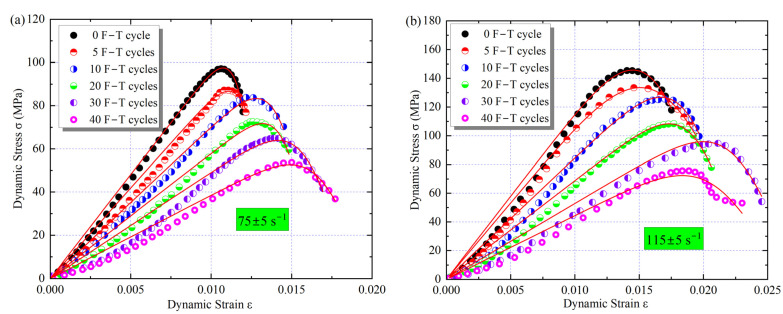
Measured and fitted stress–strain curves at different strain rates and F–T cycles. (**a**) 75 ± 5 s^−1^; (**b**) 115 ± 5 s^−1^.

**Figure 18:**
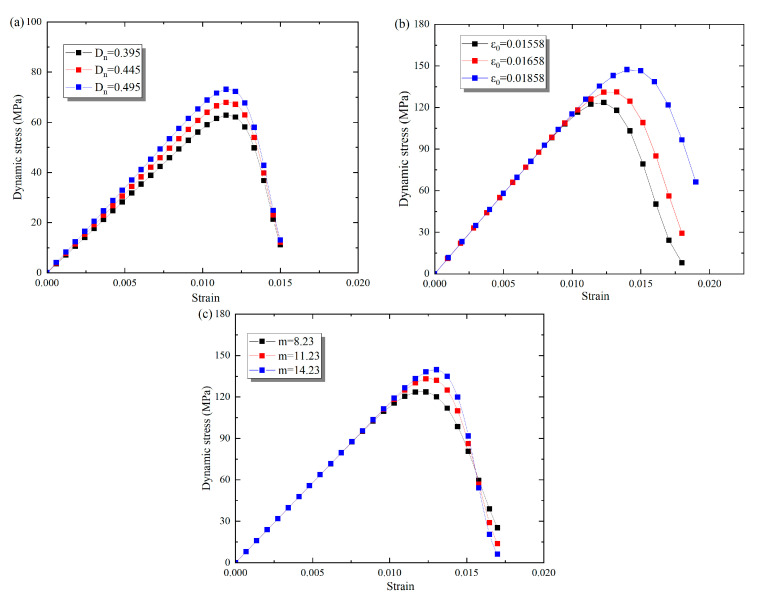
Analysis of parameter sensitivity in damage evolution model. (**a**) *D*_n_; (**b**)$\varepsilon_{0}$; (**c**) m.

The parameters *ε_0_* and *m* in the damage evolution model can be ascertained by fitting the stress–strain curves using Equation (14) (see Figure 17). The stress–strain curves under cyclic F–T effects were selected for the parametric study. As shown in Figure 18a, different *D*_n_ correspond to different stress–strain curves, and their peak stresses are inversely proportional to the parameter *D*_n_. As the name implies, the higher the damage, the lower the peak stress. Figure 18b illustrates different stress–strain relationships as *ε*_0_ is 0.0158, 0.0168, and 0.0178. The results indicate that both the peak stresses and strains increase gradually with increasing *ε*_0_. Moreover, *ε*_0_ clearly affects the trend of the descending branch of the stress–strain relationship. The stress–strain curves for different *m* are given in Figure 18c. As the *m* increases, the peak strength of the stress–strain curves increases, and the rate of stress decreases after the peak gradually increases.

**Figure 19:**
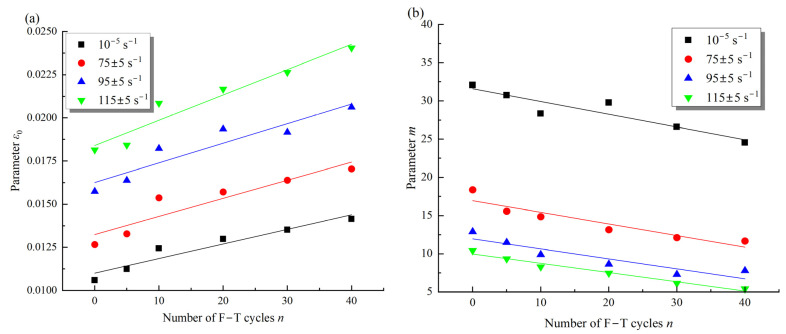
Variation of the parameters *ε_0_* (**a**) and *m* (**b**) with the strain rate and cyclic F–T action.

In addition, Figure 19 presents the variation of *m* and *ε*_0_ in the damage evolution model with the F–T action and strain rates. It can be observed that the parameter *ε*_0_ is larger under dynamic loading than under static loading, and increases with F–T damage (Figure 19a). As shown in Equation (16), the linear function can describe the change of parameter *ε*_0_ with F–T action, and the growth rate increases with the strain rates. In contrast, the parameter *m* is smaller under dynamic loading than static loading and tends to decrease linearly with the F–T action (Equation (17)). Meanwhile, the decline rate of the parameter *m* with the F–T action is inversely correlated with the strain rates, i.e., the larger the strain rate, the slower the decline rate. After five F–T cycles, the parameter *m* increased by 16% from 0.001324 to 0.001559, and the slope from 0.00105 to 0.0001123 when the strain rate was increased from 75 s^−1^ to 95 s^−1^ (Figure 19b). As the F–T cycle increases, the strength of the mineral particles and cement in the sample gradually decreases, resulting in a shift in the damage of the rock from brittle to ductile [27]. The damage of the rock also shifts from brittle to ductile under impact loading [10]. The change in damage mode implies a decrease in post-peak stress drop rate; therefore, the parameter *m* reduces with the F–T damage and loading rate. In addition, the peak strain of the sample increases with increasing F–T damage, and there is a significant strengthening effect of strain rate, so the parameter *ε*_0_ increases with the F–T action and strain rate.

(16) $$\{\begin{array}{l} \varepsilon_{0}(10^{-5}s^{-1},n)=0.011+8.45E-5\cdot n{\;(R}^{2}=0.93)\\ \varepsilon_{0}(75\pm 5s^{-1},n)=0.013+1.05E-4\cdot n{\;(R}^{2}=0.87)\\ \varepsilon_{0}(95\pm 5s^{-1},n)=0.016+1.13E-4\cdot n{\;(R}^{2}=0.95)\\ \varepsilon_{0}(115\pm 5s^{-1},n)=0.018+1.46E-4\cdot n{\;(R}^{2}=0.96) \end{array}$$

(17) $$\{\begin{array}{l} m(10^{-5}s^{-1},n)=31.61-0.167\cdot n{\;(R}^{2}=0.91)\\ m(75\pm 5s^{-1},n)=16.95-0.152\cdot n{\;(R}^{2}=0.93)\\ m(95\pm 5s^{-1},n)=11.95-0.131\cdot n{\;(R}^{2}=0.89)\\ m(115\pm 5s^{-1},n)=9.97-0.122\cdot n{\;(R}^{2}=0.96) \end{array}$$

**Figure 20:**
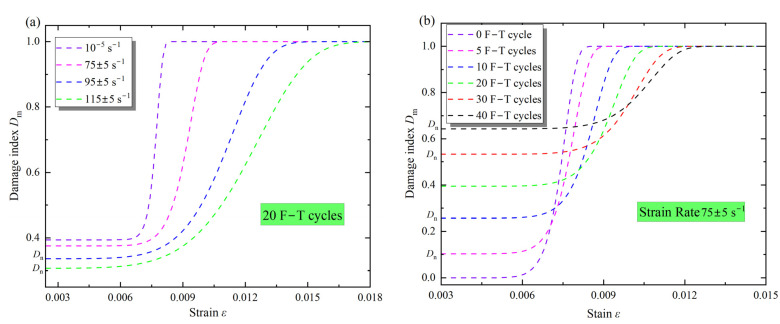
Damage *D*_m_ evolution with strain rate (**a**) and F–T cycles (**b**) under impact loading.

Based on the Equation (15) and the parameters *ε_0_*, *m*, and α, the damage evolution of the samples under dynamic loading and F–T action was investigated. The *D*_m_ with the strain rate of the samples after 20 F–T cycles is shown in Figure 20a, and the *D*_m_ with the different F–T cycles at the same loading is displayed in Figure 20b. The evolution curve of *D*_m_ exhibits distinct phase characteristics. In the initial phase, the *D*_m_ of the samples increases slowly; as the strain increases, the *D*_m_ shows a lower convex increase; when the loading continues to increase, the damage curve gradually changes to an upper convex increase; in the final phase of the stress, the rock sample fractures, and the damage curve tends to 1.

As shown in Figure 20a, the damage index *D*_m_ in the initial phase corresponds to the F–T damage *D*_n_ at different strain rates. After 20 F–T cycles, the *D*_n_ decreases with increasing strain rate, and the *D*_n_ at static loading is 0.395, which is about 1.5 times higher than that at 115 s^−1^. As the strain increases, the *D*_m_ of the sample continues to increase, but it is always between 0 and 1. This is due to the gradual development of the pores and microcracks in the rock caused by the F–T action under the loading, which eventually aggravates the damage to the sample [20]. However, compared to static loading, there are significant differences in the *D*_m_ development curves under dynamic loading: as the strain increases, *D*_m_ develops earlier in the samples with larger strain rates, e.g., at the strain rate of 115 s^−1^, damage begins at a strain of about 0.004 compared to about 0.007 under static loading. Also, the growth rate of *D*_m_ is greater under static loading, which means that the deformation during damage is less under static loading. As mentioned earlier, the peak strain of the sample increases with strain rate, leading to a decrease in the growth rate of *D*_m_ with strain in Figure 17a. At the same time, the damage mode of the samples changes from brittle to ductile under the impact loading, which is reflected in a decrease in the decrease rate of post-peak stress [20]. Therefore, the *D*_m_ of the sample under F–T action develops earlier under the impact loading, but the growth rate gradually decreases with the strain rate.

In Figure 20b, the initial strain corresponds to the different *D*_n_, and it can be observed that the *D*_n_ gradually increases with the cyclic F–T action. At the same strain rate, the evolution curves of *D*_m_ show different characteristics: The larger the *D*_n_ at the initial loading, the longer the process of slow increase of *D*_m_. This is due to the fact that the number of pores and microcracks (throats) in the sample increases continuously with the F–T action, resulting in an increase in the defect closure phase under dynamic loading [17]. With the strain increases, the pores and throats caused by the F–T action, as well as the original defects, begin to develop, so that the *D*_m_ of the sample increases in a lower convex shape [2]. As the loading continues to increase, the evolution curves of *D*_m_ gradually shift to an upper convex shape. This can be attributed to the increase in plastic strain before the peak stress with the F–T cycles, leading to a decrease in the growth rate of damage with strain. In general, the development curves of *D*_m_ changed from a sharp increase to a slow increase with strain and then to stabilization 1. The larger the strain rate, the lower the initial F–T damage, and the earlier the damage increases with strain, but the growth rate decreases with increasing strain rate; the initial damage increases gradually with the F–T action under dynamic loading, but the growth rate of damage decreases with increasing initial damage. It is worth noting that dynamic loading reduces the *D*_n_, but does not increase the growth rate of the *D*_m_.

## 5. Conclusions

The influence of microstructure on the dynamic mechanical behavior and damage evolution of frozen–thaw sandstone was investigated using the split Hopkinson pressure bar system, the MTS816 system, and the computed tomography (CT) system, revealing that microstructural change in cold regions is one of the key factors that determine the mechanical behavior of the rock under different impact loads. On this basis, a constitutive model was developed considering impact loads and F–T effects, which incorporates the influence of microscopic parameters and can well describe the stress–strain behavior under different impact loads, and provide meaningful information for understanding the dynamic hazards of rocks in cold regions.

In this study, cyclic F–T action reshapes the microstructure of the yellow sandstone, leading to an exponential increase in porosity, permeability, pore parameters, throat parameters, and fractal dimension of the samples. After 40 F–T cycles, the porosity, permeability, and fractal dimension of samples increased from 2.56, 11.3%, and 0.332 μm^2^ to 2.67, 16.7% and 0.741 μm^2^, respectively. Moreover, the peak strength and elastic modulus under impact loading were linearly and negatively correlated with microstructure, and the decrease rate was positively correlated with strain rate. Besides, with increasing impact loading, the *D*_m_ developed earlier in the samples with strain, while the growth rate of the *D*_m_ with strain was lower; with increasing F–T damage *D*_n_, the slow increase of damage was longer, and the growth rate of the *D*_m_ with strain was lower.

## Figures and Tables

**Table 1:** Mean values of physico-mechanical parameters of yellow sandstone.

V_p_ (km/s)	*ρ_d_* (g/cm^3^)	*ρ_sat_* (g/cm^3^)	*n* (%)	σ_p_ (MPa)
2623	2.14	2.27	11.3	58.7

Notes: V_p_ is the longitudinal wave velocity; *ρ_d_* is the dry density; *ρ_sat_* is the saturation density; *n* is the porosity; σ_p_ is the uniaxial compressive strength.

**Table 2:** Parameters for the Zeiss Xradia 510 Versa CT.

Voltage (kV)	Current (μA)	Exposure Time (s)	Resolution (μm)	The Number of Scan Images
100	90	5	20	1100

## Data Availability

Data available on request due to privacy restrictions.
